# Absorption peak decomposition of an inhomogeneous nanoparticle ensemble of hexagonal tungsten bronzes using the reduced Mie scattering integration method

**DOI:** 10.1038/s41598-024-57006-0

**Published:** 2024-03-19

**Authors:** Keisuke Machida, Kenji Adachi

**Affiliations:** 1 Ichikawa Research Center, Sumitomo Metal Mining Co., Ltd., Ichikawa, Chiba 272-8588 Japan; 2 Department of Product Planning and Development, Sumitomo Metal Mining Co., Ltd., 5-11-3 Shimbashi, Minato-Ku, Tokyo, 105-8716 Japan

**Keywords:** Hexagonal tungsten bronze, Nanoparticles, Near-infrared absorption, Oxygen vacancy, Ensemble inhomogeneity, Mie scattering integration, Nanoscience and technology, Optics and photonics

## Abstract

Recent optical analyses of cesium-doped hexagonal tungsten bronze have accurately replicated the absorption peak and identified both plasmonic and polaronic absorptions in the near-infrared region, which have been exploited in various technological applications. However, the absorption peaks of tungsten oxides and bronzes have not generally been reproduced well, including those of the homologous potassium- and rubidium-doped hexagonal tungsten bronzes that lacked evidence of polaronic subpeaks. The present study reports a modified and simplified Mie scattering integration method which incorporates the ensemble inhomogeneity effect and allows precise peak decomposition and determination of the physical parameters of nanoparticles. The decomposed peaks were interpreted in terms of electronic structures, screening effect, and modified dielectric functions. The analysis revealed that the plasma frequencies, polaron energies, and the number of oxygen vacancies decrease in the dopant order Cs → Rb → K. The coexistence of plasmonic and polaronic excitations was confirmed for all the alkali-doped hexagonal tungsten bronzes.

## Introduction

Tungsten bronzes possess moderately large bandgaps and abundant conduction electrons, enabling both high visible transparency and intense near-infrared (NIR) absorption^1–5^. Nanoparticles (NPs) of tungsten bronzes, in which this property is enhanced, have been employed in solar control windows^6–9^ and related applications. A growing number of studies^10–16^ are devoted to alkali tungsten bronze NPs and their applications, but the absorption mechanism and related electronic structures, including intensive optical-peak analyses, have not been sufficiently reported^4,17,18^.

Low-energy optical absorption peaks of tungsten oxides and tungsten bronzes have not been accurately decomposed thus far, although their origins are variously discussed and accepted^19–22^. Recently, however, the absorption peak profile of Cs-doped hexagonal tungsten bronze (Cs-HTB) NPs was reproduced precisely^4^ to reveal dual components involving localized surface plasmon resonance (LSPR) of free electrons and polaronic transitions of trapped electrons^3,4,20,23^. This drew attention to the isostructural alkali HTBs doped with K and Rb, as they produced no observable polaronic subpeak^6,24–26^.

**Figure 1:**
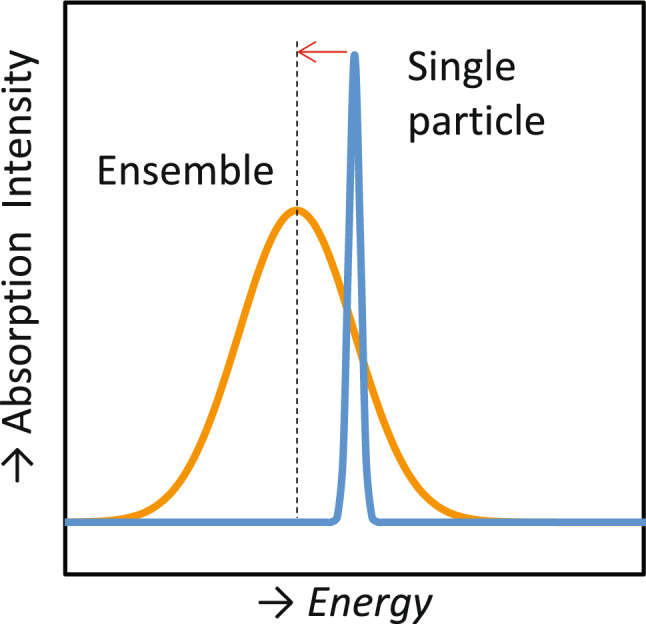
Schematic showing the ensemble inhomogeneity effect: absorption profiles of a single particle (blue) and a particle ensemble (orange).

To correctly decompose the optical peak of K- and Rb-doped HTB, one must consider the effect of the ensemble inhomogeneity^27–29^ caused by slight random variations in the particle shapes and physical properties of the individual NPs in the ensemble. Such inhomogeneity is evidenced in the large scatter in the plasmon peak energy, as directly observed in electron energy-loss spectroscopy^30^. According to optical analysis, the scatter in the plasma frequency of a NP ensemble of Cs-HTB reaches 30%^4^. Ensemble inhomogeneity is prominently observed in mechanically processed NPs, in which the particle shape, surface composition, internal oxygen vacancy (V_O_) population, and other features are modified during processing. Being composed of numerous Mie scattered waves, the absorption profile is especially sensitive to variations in particle shape and plasma frequency, which cause peak broadening and redshift as schematized in Fig. 1. In most studies, the observed profiles are described by a single Mie scatterer^31,32^, whereas an inhomogeneous ensemble actually comprises a large number of slightly different Mie scatterers. To properly treat an inhomogeneous ensemble, we previously proposed a Mie scattering integration (MSI) method with computer-generated random numbers representing spontaneous inhomogeneities^29^. This method well reproduced the complex optical profiles of plasmonic and polaronic excitations in Cs-HTB NPs and gave a unique route to determine the physical parameters of NPs in the ensemble^4,29^. However, to reproduce the experimental profiles, the original method required a special program that simulates the actual physical process involving a large number of particles (at least 1000–10,000). The present paper develops a modified and simplified MSI method that lowers the calculation load by greatly reducing the number of virtual particles. When applied to K- and Rb-HTB NPs, the new method not only provided an excellent simulation result that facilitated clarification of the absorption peak origins but also disclosed the features that distinguish K- and Rb-HTBs from Cs-HTB.

## Materials and methods

K_*x*_WO_3_, Rb_*x*_WO_3_, and Cs_*x*_WO_3_ powders (where *x* = 0.20, 0.25, 0.30, and 0.33 refers to the initial M/W weight ratio; M = K, Rb, Cs) were prepared by mixing the raw materials of alkali carbonates and tungstic acid and heating at 550 °C for 1–3 h in a 1% H_2_–N_2_ atmosphere, followed by homogenization heating at 800 °C for 1 h in a N_2_ atmosphere. The resulting deep blue powders are known to be highly reduced tungsten oxides containing many V_O_s^33^. Thus, the true chemical formulas would be K_*x*_WO_3-*y*_, Rb_*x*_WO_3-*y*_, and Cs_*x*_WO_3-*y*_, although *y* is generally ignored in the following contents.

The obtained powders were pulverized and mixed with the dispersant in methyl isobutyl ketone (MIBK) solvent. The NP-dispersion liquid was prepared in a bead mill, diluted to a fixed concentration with MIBK, and transferred to a glass holder for transmittance profile measurements in the visible and infrared wavelengths using a U-4100 spectrophotometer (Hitachi High Tech Corp., Tokyo, Japan). Powder XRD measurements were conducted using an X’Pert-PRO/MPD diffractometer (Spectris Co., Ltd., Tokyo, Japan) with Cu–Kα radiation (*λ* = 1.54 Å).

### Absorption strengths of the HTB nanoparticles

Structural characterization of the alkali HTB powders prepared was done by powder XRD and the Rietveld method. The main phase in all HTBs was hexagonal of space group *P*6_3_/mcm^34^. Trace amounts of WO_3_ and WO_2.92_ were contained in K_0.20_WO_3_, Rb_0.20_WO_3_ and Cs_0.20_WO_3_, which were found as optically negligible. All the other compositions were single phased. Further structural details including small unsymmetric strains due to pseudo Jahn–Teller distortion are reported elsewhere^35^.

**Figure 2:**
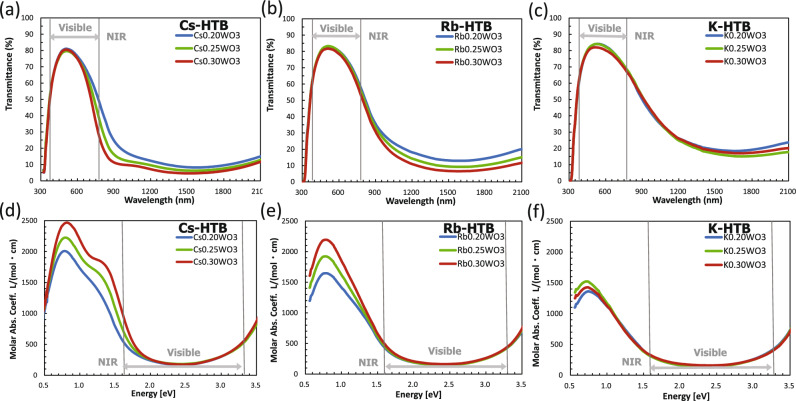
Transmission profiles (**a**–**c**) and molar absorption coefficients (**d**–**f**) of Cs_x_WO_3_, Rb_x_WO_3_ and K_x_WO_3_ (x = 0.20, 0.25, 0.30) NP dispersions with different alkali content.

The transmittance profiles of HTB NPs with nominal alkali ratios of 0.20, 0.25, and 0.30 are shown in Fig. 2a–c. All profiles exhibit a large transmission peak in the visible spectrum and a large absorption region at NIR wavelengths. However, only Cs-HTB presents a distinct dip around 850 nm. This dip was attributed to polaronic transition of localized electrons provided by V_O_s^4,23^, whereas the main broadband absorption centered around 1500 nm comprised the LSPR of free electrons activated in directions perpendicular (⊥) and parallel (ǁ) to the c-axis^4,20^.

The relative absorption strengths of the dispersed Cs-, Rb-, and K-HTB NPs were determined in terms of the molar absorption coefficient *χ*, which is defined in Lambert Beer’s law log_10_(*I/I*_0_) =  − *χ l c*, where *I*_0_ and *I* denote the intensities of the incident and transmitted light, respectively, *l* is the optical length (cm), and *c* is the molar concentration (mol/L). The absorption coefficient of the bulk is inapplicable to a NP dispersion, whereas the commonly used absorbance or transmittance is inaccurate as a measure because the filler concentration is unknown. For these reasons, we selected *χ* as a measure of the absorption strength^6^. The *χ* of a solution containing 1 mol of NP filler must be measured under the same Mie scattering conditions, i.e., the same particle shape and size, same degree of dispersion (particle distribution), and same refractive constant of the medium. These constraints are crucial because the solute in the present case is not a homogeneous dye but discrete NPs that undergo Mie scattering and LSPR.

Figure 2d**–**f presents the χs corresponding to Fig. 2a**–**c. The χs were measured under the same Mie scattering condition with the particle size within 25–30 nm. All *χ* profiles exhibit an absorption peak between 0.5 and 2.0 eV. The peak sizes considerably decreased as Cs → Rb → K, which cannot be deduced from Fig. 2a**–**c unless the filler amounts are specified. The absorptions of all HTB NPs in Fig. 2d**–**f decreased as the doping amount decreased from M/W = 0.30–0.20, although the small decrement suggests that the dopant should not be the only source of the absorption. Moreover, the magnitude of the decrement reduced from Cs-HTB to K-HTB (where K_0.25_WO_3_ and K_0.30_WO_3_ were reversed either by error or by an unclarified reason. See ref.^35^). If the alkali dopant alone caused the NIR absorption^36–38^, such changes are not expected. Therefore, both the dopant-donated and V_O_-donated electrons must be involved in the low-energy absorption.

The absorption peak of Cs-HTB in Fig. 2d comprises the main peak at 0.8 eV and a subpeak at 1.4 eV, whereas the subpeaks of Rb- and K-HTB are not clearly defined^6,24–26^. The absorption peaks were decomposed using Mie theory with consideration of the ensemble inhomogeneity. In previous work^29^, we examined the effects of particle size distribution and particle shape on absorption peaks of Cs-HTB dispersions using TEM and small-angle x-ray scattering and concluded that their effects were negligible as compared to those due to the variations in dielectric functions. So, the optical profiles of Cs_x_WO_3-y_ (0.20 ≤ *x* ≤ 0.33, 0.17 ≤ *y* ≤ 0.46) NPs were decomposed by integrating 10,000 Mie scattered waves from randomly computer-generated particles, each having a different plasma frequency and polaron energy^4,29^. To avoid large-scale calculations, we here assume a normal distribution of parameters defining the absorptions. In addition, we replace the complex arithmetic of Mie scattering by a simple formula under the quasistatic approximation^39^. These simplified procedures are described in the next section.

### Simplified procedure of the Mie scattering integration method

NIR absorption from a single NP of an alkali HTB is contributed by LSPR⊥ and LSPRǁ of free electrons and polaronic transition of trapped electrons^2,4,35^. The optical behavior of free electrons is described by the Drude oscillator in the free electron model, whereas the interband transitions of trapped electrons is described by the Lorentz oscillator. Thus, we assume the Drude and Lorentz terms in the dielectric function (in cgs unit) as follows:

1 $${\upvarepsilon } = {\upvarepsilon }_{\infty } - \frac{{{\Omega }_{{\text{p}}}^{2} }}{{{\upomega }^{2} + {\text{i}}\upomega \upgamma }} + \frac{{{\Omega }_{{\text{E}}}^{2} }}{{{\upomega }_{{\text{T}}}^{2} - {\upomega }^{2} - {\text{i}}{\upomega \Gamma }}},$$

2 $${\Omega }_{{\text{p}}}^{2} = \frac{{4\pi Ne^{2} }}{{m^{*} }},$$

where *ε*_∞_, Ω_p_, and *γ* in the Drude term, ${\varepsilon }_{\infty }- \frac{{{\Omega }_{{\text{p}}}^{2} }}{{{\upomega }^{2} + {\text{i}}\upomega \upgamma }}$, denote the high-frequency permittivity, plasma frequency, and relaxation constant, respectively, and *ω*_T_, Ω_E_^2^, and Γ in the Lorentz term denote the peak frequency, peak area, and peak width, respectively. The plasma frequency is expressed by Eq. (2), where *N* and m* denote the carrier density and effective mass, respectively.

The dielectric function is assumed to reduce to the Drude term at low energies^1^. Under the quasistatic approximation, the Mie extinction cross-section is given by^31^

3 $$\begin{array}{*{20}c} {\sigma_{ext} = A\frac{1}{\lambda }\text{Im}\left( {\frac{{\varepsilon - \varepsilon_{m} }}{{\varepsilon + 2\varepsilon_{m} }}} \right),} \\ \end{array}$$

where *λ* is the wavelength, *ε*_m_ is the permittivity of the surrounding medium, and *A* is a constant. Im(*z*) indicates the imaginary part of the complex number *z*. The quasistatic approximation is valid when 2π*ε*_m_*d*/*λ* << 1 (*d* = particle size)^31^, thus applicable to a present particle of *d* approximately less than 30 nm in MIBK of *ε*_m_ = 2.25. Substituting the Drude term of Eq. (1) into Eq. (3), we can express the LSPR absorption in terms of Drude parameters alone as follows:

4 $$\sigma_{ext}^{LSPR} = \frac{{B\gamma \Omega_{p}^{2} \omega^{2} }}{{\left( {\varepsilon_{\infty } + 2\varepsilon_{m} } \right)^{2} \omega^{4} + \left( {\varepsilon_{\infty } + 2\varepsilon_{m} } \right)\left[ {\left( {\varepsilon_{\infty } + 2\varepsilon_{m} } \right)\gamma^{2} - 2\Omega_{p}^{2} } \right]\omega^{2} + \Omega_{p}^{4} }},$$

where *B* is a constant.

Reflecting the crystalline anisotropy in HTB, the Drude parameters have components perpendicular (⊥) and parallel (ǁ) to the *c*-axis^2^. Therefore, the Drude factor involves six parameters: $\varepsilon_{\infty \bot }$, ${\Omega }_{{{\text{p}} \bot }}$, $\gamma_{ \bot }$, $\varepsilon_{\infty \parallel }$, ${\Omega }_{{{\text{p}}\parallel }}$ and $\gamma_{\parallel }$.

The absorption due to polaronic excitation is incorporated via the Lorentz term in Eq. (1). Alternately, it may be directly expressed by the extinction cross section, quoting from Austin and Mott^22,40^,

5 $$\begin{array}{*{20}c} {\sigma_{ext}^{polaron} = \frac{{A_{p} }}{\hbar \omega }\exp \left[ { - \frac{{\left( {\hbar \omega - 2E_{B} } \right)^{2} }}{{8E_{B} k\tau }}} \right],} \\ \end{array}$$

where *A*_p_ is a temperature-dependent constant, *ħ* is reduced Planck’s constant, *E*_B_ is the binding energy, *k* is the Boltzmann constant, and *τ* is the absolute temperature (K). Equation (5) was derived for small polarons by contributions of Reik and Heese^41^, Bogomolov et al.^42^ and others, and reviewed by Austin and Mott^40^. Small polarons were suggested in Cs-HTB, as the Voronoi local charge calculation revealed the extra charges on W ions adjacent to V_O_^5^. As both expressions of the polaronic term involve three parameters, i.e., *ω*_T_, Ω_E_, and *Γ* for the Lorentz expression and *A*_p_, *E*_B_, and *τ* for the Austin–Mott expression, nine parameters must be described for each particle. Below, we illustrate the MSI procedure with the Austin–Mott expression.

The total extinction cross-section of an *N*-particle ensemble is given by:

6 $$\begin{array}{*{20}c} {\sigma_{ext} = \mathop \sum \limits_{X} \left\{ {\sigma_{ext}^{LSPR \bot } + \sigma_{ext}^{LSPR\parallel } + \sigma_{ext}^{polaron} } \right\},} \\ \end{array}$$

where *X* is a general term for the parameters involved in the Drude, Lorentz, or Austin–Mott terms (each particle has nine independent *X*s). Correlations between *X*s of different particles can be neglected in the present dilute system, as previously shown by measuring *σ*_ext_ for dispersions of different inter-particle distances^29^. We also assume no correlations between *X*s inside a particle. An actual single NP has both aspects of LSPR⊥ and LSPRǁ, which we treat mathematically as 2/3 particles in the ensemble oriented such as to activate only LSPR⊥ and 1/3 particles only LSPRǁ (Thus, coefficient B of $\sigma_{ext}^{LSPR \bot }$ is set around twice that of $\sigma_{ext}^{LSPR||}$). This treatment assumes no correlation, but explains the experimental curves very well, as shown later.

Let us introduce a uniform variation of *X* given by *X*_M_ ± 3.0*s*_X_, where *X*_M_ and *s*_X_ denote the mean (central) value and standard deviation of *X*, respectively. The total number of values in *X* can be an arbitrary odd number greater than (say) 10 and less than 100, depending on the nature of the ensemble. If the number is too small, calculated *σ*_ext_ profiles do not fit to experimental values. As the number increases, they converge to a fixed fitted curve. Here, we illustrate with 61 particles labeled No. 0 to No. 60. The value of *X* of particle No. *i*, *X*_*i*_, is then given by:

7 $$X_{i} = X_{M} + \left( {0.1i - 3.0} \right)s_{x} .$$

Here, the parameters of particles No. 0, 30, and 60 are given by $X_{0} = X_{M} - 3.0s_{x} , X_{30} = X_{M}$ and $X_{60} = X_{M} + 3.0s_{x} ,$ respectively. That is, every parameter takes a value within ± 3.0*s*_X_ from the mean value with a uniform interval. The value of *s*_X_ is restricted to be within 1/3 (33%) because the parameter values must be positive ($X_{0} = X_{M} - 3.0s_{x}$ > 0).

**Figure 3:**
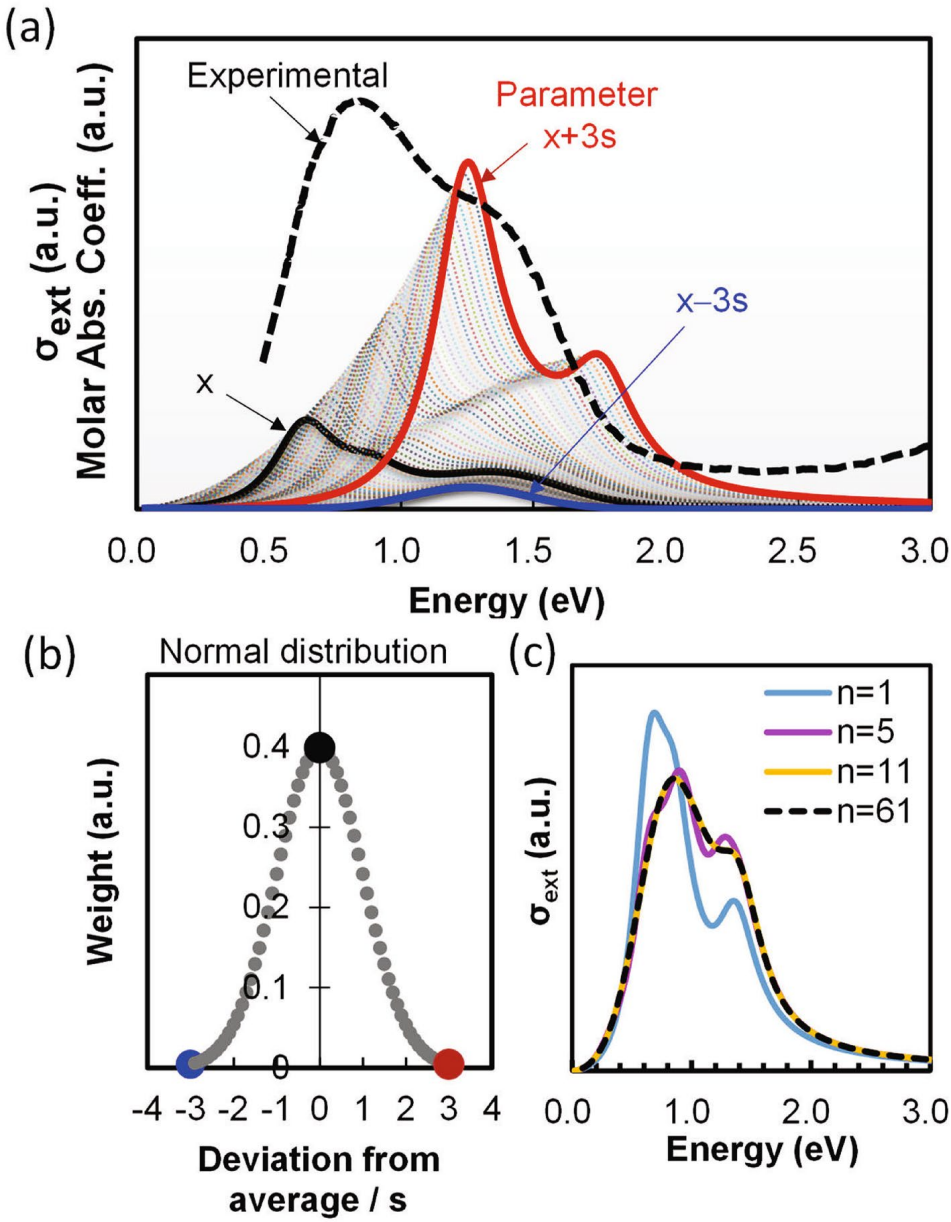
(**a**) Extinction profiles (dotted lines) of 61 virtual particles with scattered dielectric parameters. The experimental profile (dashed line) is also shown for comparison. (**b**) Assumed occurrence-ratio distribution of the extinction profiles. (**c**) Extinction profiles calculated for 1, 5, 11, and 61 virtual particles.

The extinction profiles of the 61 particles calculated by Eq. (6) are shown in Fig. 3a. The experimental molar absorption coefficient is plotted in the same chart (note that the extinction reduces to absorption in our particle sizes of concern, as the scattering becomes negligible). After summing these 61 extinction profiles, we expect a large discrepancy from the experimental profile. In the actual distribution, however, the probability of occurrence decreases with increasing deviation of the parameter from its mean value. Therefore, we assign a normally distributed weighting multiplier $p_{i}$ to particle No. *i* (Fig. 3b):

8 $$p_{i} = \frac{1}{{\sqrt {2\pi } }}\exp \left[ { - \frac{{\left( {0.1i - 3.0} \right)^{2} }}{2}} \right] .$$

The *σ*_ext_ of absorption factor *Y* (*Y* = LSPR⊥, LSPRǁ, or polaron) is assumed to depend only on *X*. It is calculated as

9 $$\begin{array}{*{20}c} {\sigma_{ext}^{Y} = \mathop \sum \limits_{i = 0}^{60} p_{i} \sigma_{ext,i}^{Y} \left( {X_{i} } \right).} \\ \end{array}$$

Among the nine parameters, we varied the most significant and decisive parameters (namely, ${\Omega }_{{{\text{p}} \bot }}$, ${\Omega }_{{{\text{p}}\parallel }}$ and $E_{B}$^29^) and the other parameters were determined as the best fit values through a trial and error. The total extinction cross-section is generally expressed as

10 $$\sigma_{ext} = \mathop \sum \limits_{i} p_{i} \sigma_{ext,i}^{LSPR \bot } \left( {{\Omega }_{p \bot ,i} } \right) + \mathop \sum \limits_{j} q_{j} \sigma_{ext,j}^{LSPR\parallel } \left( {{\Omega }_{p\parallel ,j} } \right) + \mathop \sum \limits_{k} r_{k} \sigma_{ext,k}^{polaron} \left( {E_{B,k} } \right).$$

The summations over *i*, *j*, and *k* are independent and can be simplified to a summation over *i*. The total extinction cross-section from the 61 NPs, which will be fitted to the experimental curves, is then given by:

11 $$\sigma_{ext} = \mathop \sum \limits_{i = 0}^{60} p_{i} \sigma_{ext,i}^{LSPR \bot } \left( {{\Omega }_{p \bot ,i} } \right) + \mathop \sum \limits_{i = 0}^{60} p_{i} \sigma_{ext,i}^{LSPR\parallel } \left( {{\Omega }_{p\parallel ,i} } \right) + \mathop \sum \limits_{i = 0}^{60} p_{i} \sigma_{ext,i}^{polaron} \left( {E_{B,i} } \right).$$

On executing the analysis, the successful range of *s*_X_ is 1/10–1/3, depending on the nature of the ensemble and the extent of parameter inhomogeneities. In the present ensemble, $s_{x}$ of ${\Omega }_{{{\text{p}} \bot }}$, ${\Omega }_{{{\text{p}}\parallel }}$ and $E_{B}$ was taken to be 1/3. Note also that *ε*_∞_ must be greater than unity. In the course of our trials, peak decompositions using Eq. (11) generally gave unique results within a narrow range without imposing any a priori conditions. In our previous analysis^4,29^, the MSI fitting was set under two constraints, fixing the ${\Omega }_{p \bot }$/${\Omega }_{p||}$ ratio to 5.11/3.09 and taking the same bandwidth for LSPR⊥ and LSPRǁ. However, these constraints were found not just necessary but could possibly lead to slight deviation from the true value if strong screening effect (see below) occurred between peaks. Notably, this MSI method requires no information on the filler quantity throughout the analysis; the useful physical parameters of the NPs are obtained simply from the transmittance or absorbance data of the NP dispersions.

### Analysis of the absorption peaks of HTB NPs

**Figure 4:**
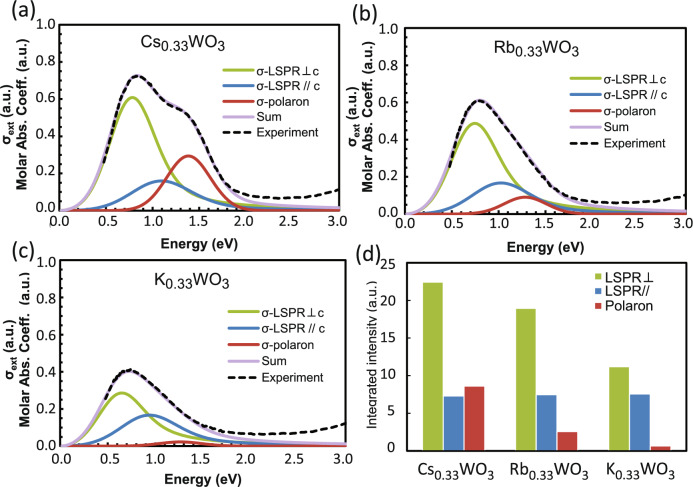
Molar absorption coefficients of (**a**) Cs_0.33_WO_3_, (**b**) Rb_0.33_WO_3_ and (**c**) K_0.33_WO_3_ NPs decomposed into the LSPR⊥c, LSPRǁc, and polaron components and (**d**) their peak area fractions.

The Mie scattered waves were integrated such that the total *σ*_*ext*_ of the ensemble given by Eq. (11) fitted the experimental absorption profiles. The shape of calculated profiles gradually changed and converged to a fixed one as the number of virtual particles increased, as shown in Fig. 3c. It suggests that as low as 11 particles may be sufficient to calculate for this ensemble, though below we present the result with 61 particles. As shown in Fig. 4, the molar absorption coefficients were successfully decomposed into their three main components, yielding numerical values of Ω_p_, γ, ε_∞_, *E*_B_, *A*_p_, Ω_E_, *Γ*, ω_T_ and *P* (see Table 1). A small discrepancy seen at energies greater than 2.0 eV arises from the band-edge absorption around 3.5 eV and slight interband excitations due to the insufficient bandgap between 2.0 and 3.5 eV. The results of the simplified method almost matched those of the original MSI method, as shown for Cs_0.32_WO_3_ in parentheses in Table 1.

**Table 1:** Plasmon and polaron parameter values obtained by decomposing the optical absorption profiles of the NPs.

	Cs_0.32_WO_3_	Rb_0.33_WO_3_	K_0.33_WO_3_
LSPR1			
ħΩ_p_⊥ (eV)	1.69 (1.64)	1.60	1.38
ħγ⊥ (eV)	0.29 (0.31)	0.35	0.40
ε_∞_⊥	2.1 (1.8)	2.1	2.1
P⊥ (eV)	0.78 (0.78)	0.74	0.64
LSPR2			
ħΩ_p_*‖* (eV)	3.00 (3.00)	2.80	2.55
ħγ‖ (eV)	0.30 (0.30)	0.32	0.38
ε_∞_‖	6.0 (6.0)	6.0	6.0
P‖ (eV)	1.10 (0.96)	1.02	0.94
Polaron (Austin‒Mott)			
A_P_ (eVcm^2^)	0.42 (0.33)	0.12	0.03
E_B_ (eV)	0.71 (0.72)	0.66	0.65
P_pol_ (eV)	1.38 (1.40)	1.28	1.26
Polaron (Lorentz)			
ħΩ_E_ (eV)	0.85 (0.58)	0.45	0.30
ħΓ (eV)	0.40 (0.50)	0.40	0.40
ħω_T_ (eV)	1.33 (1.35)	1.30	1.15

The polaron component was calculated using both the Austin–Mott and Lorentz expressions. Values of Cs-HTB in parentheses are those using the original MSI method^4^.

The important trend in Table 1 is the shift in Ω_p_, *P*, *E*_B_, Ω_E_, and *P*_pol_ toward lower energy in the order of Cs → Rb → K, reflecting a consistent decrease in free and localized electrons. This indicates that the amount of V_O_ should decrease in the same dopant order because the alkali content was fixed at *x* = 0.33. Chemical and XRD Rietveld analyses of sintered bulk HTBs yielded the same results of the decreasing V_O_ as Cs → Rb → K^35^.

**Figure 5:**
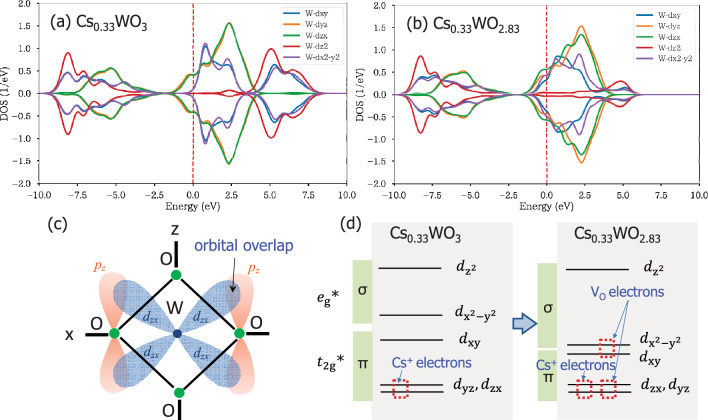
Calculated densities of states in the W‒*d* orbitals of (**a**) V_O_-free Cs_0.33_WO_3_ and (**b**) V_O_-incorporated Cs_0.33_WO_2.83_ [quoted from Ref.^43^]; (**c**) schematic of W‒*d* and O‒*p* hybridized orbitals in a WO_6_ octahedron projected onto the *x‒z* plane; (**d**) molecular orbital diagrams of HTB^43^ showing the W‒*d* orbitals accommodating the V_O_- and Cs-donated electrons.

To understand the triplet peaks of LSPR⊥, LSPRǁ and polarons in Fig. 4a–c, the electronic structures of HTB are quoted^43^ in Fig. 5. According to first-principles calculations^5,43^, the alkali-donated electrons occupy the W‒*d*_yz_ and *d*_zx_ orbitals (Fig. 5a) while the V_O_-donated electrons occupy the W − ${d}_{xy}$, ${d}_{{x}^{2}-{y}^{2}}$ orbitals in addition to the W‒*d*_yz_ and *d*_zx_ orbitals (Fig. 5b). These W‒*d* electrons are hybridized with the O‒*p* orbital electrons (Fig. 5c) to construct *t*_2g_* and *e*_g_* states in the π and σ bonds, respectively (see the molecular orbital diagrams in Fig. 5d). Herein, the geometrical electronic distributions across the overlapped orbitals suggest that the W − ${d}_{xy}$, ${d}_{{x}^{2}-{y}^{2}}$ electrons in *e*_g_* are related only to LSPR⊥, whereas the W − *d*_yz_ and *d*_zx_ electrons in *t*_2g_* are related to both LSPR⊥ and LSPRǁ. Thus, the LSPR⊥ peak should be contributed by both the V_O_ and Cs electrons in W − ${d}_{xy}$, ${d}_{{x}^{2}-{y}^{2}}$, *d*_yz_ and *d*_zx_, whereas the LSPRǁ peak should be contributed by the Cs and V_O_ electrons in W − *d*_yz_ and *d*_zx_. The polaron peak is derived entirely from the V_O_ electrons because it did not emerge without the presence of V_O_ even if the dopants existed^4,5^. Hence, the intensities of the LSPR⊥ and polaron peaks decreased in the dopant order of Cs → Rb → K, as expected (Fig. 4d). On the other hand, the LSPRǁ peak remained nearly unchanged, even though it is contributed by both the Cs and V_O_ electrons.

**Figure 6:**
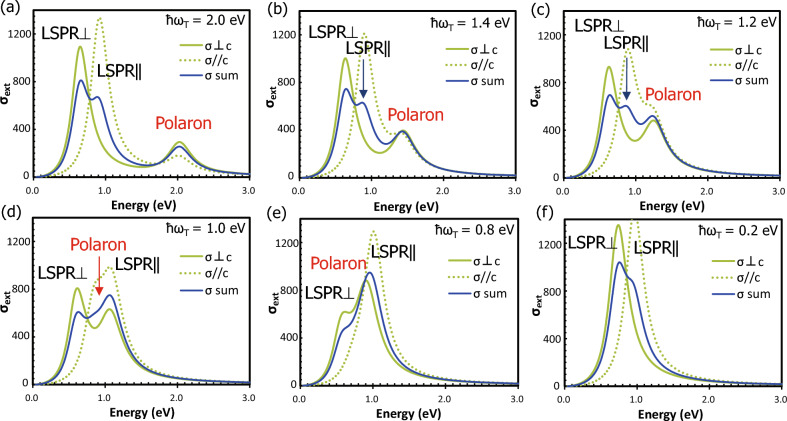
The electron screening and anti-screening effects of contiguous excitations calculated for the different locations of polaron peak with respect to LSPR⊥ and LSPRǁ peaks. Parameter values used are ε_∞⊥_ = 2.1 eV, Ω_p⊥_ = 1.69 eV, γ_⊥_ = 0.29 eV, ε_∞ǁ_ = 6.0 eV, Ω_pǁ_ = 3.00 eV, γ_ǁ_ = 0.30 eV, Ω_E_ = 0.85 eV, Γ = 0.40 eV, and ω_T_ = varied.

To discuss the peak intensities, one needs additionally to inspect the electron screening effect for contiguous excitations^4,44^. A typical behavior of absorption peaks due to this effect is simulated as shown in Fig. 6. For a given set of anisotropic LSPR peaks, the interband transition (polaron) peak was postulated to approach from the high energy side by varying ħ*ω*_T_. Parameter values used are *ε*_∞⊥_ = 2.1, ħΩ_p⊥_ = 1.69 eV and ħ*γ*_⊥_ = 0.29 eV for LSPR⊥, *ε*_∞ǁ_ = 6.0, ħΩ_pǁ_ = 3.00 eV and ħ*γ*_ǁ_ = 0.30 eV for LSPRǁ, and ħΩ_E_ = 0.85 eV and ħ*Γ* = 0.40 eV for the polaron peak, respectively. Here, the extinction cross section, *σ*_ext_, was calculated under quasistatic approximation^39^ using Eq. (3). The anisotropic *σ*_⊥_ and *σ*_ǁ_ were separately calculated and averaged with weights by $\sigma =(2{\sigma }_{\perp }+{\sigma }_{\parallel })/3$. When the polaron peak approaches the LSPR⊥ and LSPRǁ peaks from the high energy side, both the LSPR peaks decrease their intensities and are pushed toward the low energy side (screening). When ħ*ω*_T_ falls behind the LSPRǁ peak energy at around 1.1 eV, the relative locations are interchanged and the LSPRǁ peak is intensified (anti-screening). Therefore, the screening effect of K-HTB with less V_O_ and weaker polaron peak increases the relative peak intensity of LSPR⊥ and LSPRǁ, which explains the behavior of LSPRǁ peaks in Fig. 4d. It should be noted that the screening effect of nearby peaks can be automatically incorporated in *σ*, as done in Fig. 6, if one includes the polaron term in Eq. (6) not as ${\sigma }_{ext}^{polaron}$ but in the expressions of ${\sigma }_{ext}^{LSPR\perp }$ and ${\sigma }_{ext}^{LSPR\parallel }$ by using anisotropic *ε*_⊥_ and *ε*_ǁ_ that include the polaron term. This procedure was adopted in our previous work^4^.

Returning to the triplet peaks in Fig. 4, it has been clarified^29,32^ that, for both plasmonic and polaronic absorptions, the peak gets weakened and peak position redshifted when V_O_ is decreased. Therefore, it should be the relative redshift and diminution of the polaron peak with respect to the decreased plasmon peak, which caused the less prominent polaronic shoulder in Rb- and K-HTBs, in contrast to the distinct shoulder observed in Cs-HTB. The large difference in χ of M-HTBs as shown in Fig. 2d**–**f is now clearly resolved as attributed to the difference in the V_O_ amount involved.

**Figure 7:**
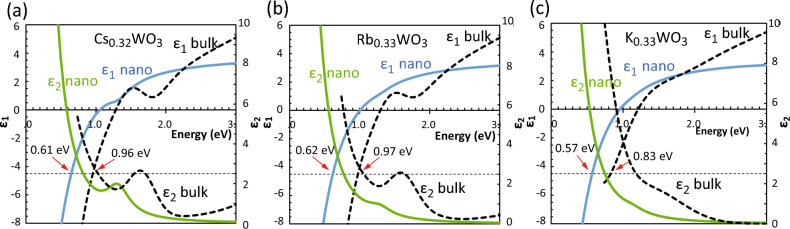
Effective dielectric functions of (**a**) Cs_0.33_WO_3_, (**b**) Rb_0.33_WO_3_ and (**c**) K_0.33_WO_3_ NPs at low energies, showing significant modifications from their respective bulk dielectric functions. Red arrows indicate the screened plasma frequencies.

Table 1 also reveals changes in the physical properties of NPs relative to the bulk properties. For instance, Ω_p_ is notably smaller in the NPs than in bulk crystals (bulk data given in^35^). Figure 7 compares the dielectric functions of a single NP derived from the data in Table 1 with those in bulk crystals. The screened plasma frequencies at *ε*_1_ =  − 4.5 in the *ε*_1_ profiles of the NPs dispersed in MIBK (*ε*_m_ = 2.25) are considerably decreased from those of bulk HTBs, from 0.96 to 0.61 eV in Cs-HTB NPs, from 0.97 to 0.62 eV in Rb-HTB NPs, and from 0.83 to 0.57 eV in K-HTBs NPs. The polaron peak in the *ε*_2_ profile is also substantially diminished and redshifted in the NPs relative to bulk. These deteriorations of 31–36% in the dielectric properties are naturally attributed to the decrease in alkali ions on the particle surfaces^7,45^ and the decrease in interior V_O_s^29^ during the milling process. These clarifications show that MSI analysis for peak decomposition is highly useful in analyzing the physical properties of a NP in the ensemble without incurring a heavy calculation load. The present method should also be applicable to the optical analyses of various ensembles including noble metal colloids, chalcogenide and perovskite quantum dots, and metal oxide nanoparticle dispersions, if their functional model is known.

## Conclusions

The optical absorption profiles of reduced K-, Rb-, and Cs-HTB NPs were decomposed using the modified MSI method and analyzed in terms of the dopant and V_O_ electrons. The number of virtual particles to be integrated was considerably lowered by approximating the parameter variations as normal distributions. The new calculation scheme incorporated only 61 particles with varied parameters and its results minutely differed from those of the original method, which was rigorously executed with 10,000 particles. It was suggested that number of virtual particles may be minimized to 11 in the present ensemble.

The new method decomposes the absorption peaks into two LSPR and one polaronic excitation, as previously analyzed for Cs-HTB^4^. In NPs of all the alkali HTB, free electrons coexisted with trapped electrons having similar energies. The plasma frequencies and polaron energies of NPs decreased in the dopant order Cs → Rb → K, consistent with the V_O_ decrease observed in sintered bulk plates^35^. It was found that the LSPR⊥ peak is derived from Vo electrons in W−${d}_{xy}$ and W−${d}_{{x}^{2}-{y}^{2}}$ orbitals and Cs electrons in W‒*d*_yz_ and W‒*d*_zx_, whereas the LSPRǁ peak is derived from Cs and V_O_ electrons in W‒*d*_yz_ and W‒*d*_zx_ orbitals. The decomposed peak location and intensity were consistently interpreted in terms of the electronic structures, screening effect, and modified dielectric functions. The decreased and redshifted LSPR⊥ and polaron peaks, combined with the relatively stable LSPRǁ peak, caused the shoulder-less appearance of the absorption peaks of the K- and Rb-HTBs NPs. The decomposition of the absorption peaks also enabled to determine the effective dielectric functions and physical parameters of an average NP in the ensemble. V_O_s largely influenced the optical properties of the alkali HTBs, and their effect increased in the dopant order K → Rb → Cs. The validity of the present MSI method is not limited to the present HTB materials but may be extended to other plasmonic and polaronic systems, including noble metal, metal oxide, chalcogenide, and perovskite NPs.

## Data Availability

The datasets used and/or analyzed during the current study are available from the corresponding author on reasonable request.
